# Exploring the efficacy and safety of herbal medicine on Korean obese women with or without metabolic syndrome risk factors: A study protocol for a double-blind, randomized, multi-center, placebo-controlled clinical trial

**DOI:** 10.1097/MD.0000000000021153

**Published:** 2020-07-10

**Authors:** Youme Ko, Hyun-Ju Kim, Hojun Kim, Jin-Bong Choi, Young-Dal Kwon, Won-Seok Jung, Bo-Hyoung Jang, NamKwen Kim, Yun-Kyung Song, Seong-Gyu Ko

**Affiliations:** a Department of Korean Preventive Medicine, College of Korean Medicine, Kyung Hee University, Seoul; b Institute of Safety and Effectiveness Evaluation for Korean Medicine, Kyung Hee University, Seoul; c Oriental Medicine Research Institute, College of Korean Medicine, Gachon University, Seongnam; d Department of Korean Rehabilitation Medicine, College of Korean Medicine, Dongguk University, Seoul; e Department of Oriental Rehabilitation Medicine, Gwangju Oriental Hospital of Dongshin University, Gwangju; f Department of Korean Medicine Rehabilitation, Gwangju Medical Center, College of Korean Medicine, Wonkwang University, Gwangju; g Department of Korean Rehabilitation Medicine, College of Korean Medicine, Kyung Hee University, Seoul; h Center for Comparative Effectiveness Research & Economic Evaluation in Korean Medicine, Pusan National University, Yangsan; i Department of Korean Rehabilitation Medicine, College of Korean Medicine, Gachon University, Seongnam, Republic of Korea.

**Keywords:** Galgeun-tang, Korean medicine, metabolic syndrome, obesity, risk factors

## Abstract

**Background:**

The prevalence of obesity among women is increasing. Obesity is associated with various metabolic syndromes; conventional treatments are limited and may induce serious adverse events due to polytherapy regimens. Currently, demands for complementary and alternative medicine that has a proven safety profile for the treatment of obesity with or without metabolic risk factors are increasing.

Our team of preclinical experts reported a significant anti-obesity effect of the Korean herbal medicine, Galgeun-tang (GGT). Thus, we designed this trial to explore the effects of GGT among obese women to accumulate optimal clinical evidence.

Obesity is not only a component of metabolic syndrome and a factor associated with an increased risk of cardiovascular disease but is also related to insulin resistance. Previous research has confirmed that an increasing body mass index is highly related with increased risk of metabolic syndrome among overweight and obese individuals. The effectiveness of the Korean medicine herbal formula, GGT on obesity has been previously reported. The objective of this study is to assess the efficacy and safety of GGT for weight loss among obese Korean women with or without high risk for metabolic syndrome.

**Methods/design:**

This study is a randomized, double-blinded, placebo-controlled, multi-center clinical trial. A total of 160 participants will be randomly distributed in 2 groups, the GGT group or the placebo group in a 1:1 ratio using a web-based randomization system. Each group will be administered GGT or placebo 3 times a day for 12 weeks. The primary endpoint is to assess the change in weight from baseline. The secondary endpoints are the following: the changes in body composition measurements, anthropomorphic measurements, obesity screening Laboratory tests, patient self-reported questionnaires, and economic evaluation outcomes. Adverse events will also be reported.

**Discussion:**

The findings of this study will confirm methodologies regarding the efficacy and safety of GGT for weight loss among obese Korean women with or without metabolic risk factors.

## Abstract 2

Supplemental Digital Content is available in the text

## 1 Introduction

The current increase in the prevalence of obesity is considered a serious major health problem among the female population. Currently, the National Cholesterol Education Program Adult Treatment Panel-III guideline identifies that obesity is one of the components of metabolic syndrome which is related to cardiovascular disease.^[1]^ According to a previous study of the Third National Health and Nutrition Examination Survey, the increasing prevalence of obesity-related comorbidities such as type 2 diabetes mellitus (T2DM), hypertension, and osteoarthritis may lead to increase the severity of the effects of being overweight or obese among adults aged 25 and older in the United States.^[2]^ Based on the data from Korea National Health and Nutrition Examination Survey the prevalence of obesity in Korea is steadily rising from 30.7% in 2008 to 34.6% in 2018.^[3]^ In addition, according to a study of the socioeconomic burden of obesity in Korea, over 11.4 trillion won was spent due to obesity in 2016. Among the costs for medical expenditures, diabetes accounted for 22.6%, followed by hypertension at 21.6%, ischemic heart diseases at 8.7%, and arthropathy at 7.8%.^[4]^ Thus, obesity and metabolic risk factors are a disease burden that warrants prevention to reduce the likelihood of various complications.

Currently, complex medication regimens are a growing concern in patient care.^[5]^ Due to the complex mechanism of obesity associated with the components of metabolic syndrome, polytherapy is widely practiced as a treatment regimen for obesity and obesity-related comorbidities.^[6,7]^ According to previous reports, an increased number of prescribed medications can decrease drug adherence and increase the risk of various adverse effects.^[8,9,10]^ Because of the potential harmful consequences, the demands for new alternative treatments are increasing, and in connection with this, a variety of herbal medicine-based drugs that control weight and overweight related conditions with proven safety compared to conventional medicines are gradually emerging.^[11,12]^

In Korean medicine, Galgeun-tang (GGT; Galgeun-tang in Chinese and Kakkon-to in Japanese), a traditional herbal prescription that originated from <Treatise on Exogenous Febrile Disease>, has been used widely for the treatment of the common exogenous symptoms, such as cold, muscle stiffness, pain, and fever.^[13]^

Several recent in vivo and in vitro studies of GGT have reported antiviral, anti-inflammatory, anti-viral, anti-allergic, antioxidant, and anti-adipogenic effects of GGT.^[14–20]^ Ki et al reported that GGT significantly decreased levels of glucose and triglyceride (TG), total body weight, liver and fat weight in an obese animal model of hyperlipidemia and diabetes. Further, GGT also increases high density lipoprotein (HDL) levels.^[21]^ However, there is currently a lack of clinical evidence in the antiobesity effect of GGT among Korea women. Thus, we plan to conduct a clinical trial on the safety and efficacy of GGT among obese women with or without other metabolic risk factors.

## 2 Study objectives

The primary objective is to assess the safety and efficacy of 12 weeks of treatment with GGT among obese Korean women with or without metabolic syndrome risk factors compared to a placebo group. Our additional intent is to provide a basis for listing health insurance benefits for this treatment by performing a cost-effectiveness analysis through long-term follow-up observation of the proven efficacy.

## 3 Study design

**Figure 1:**
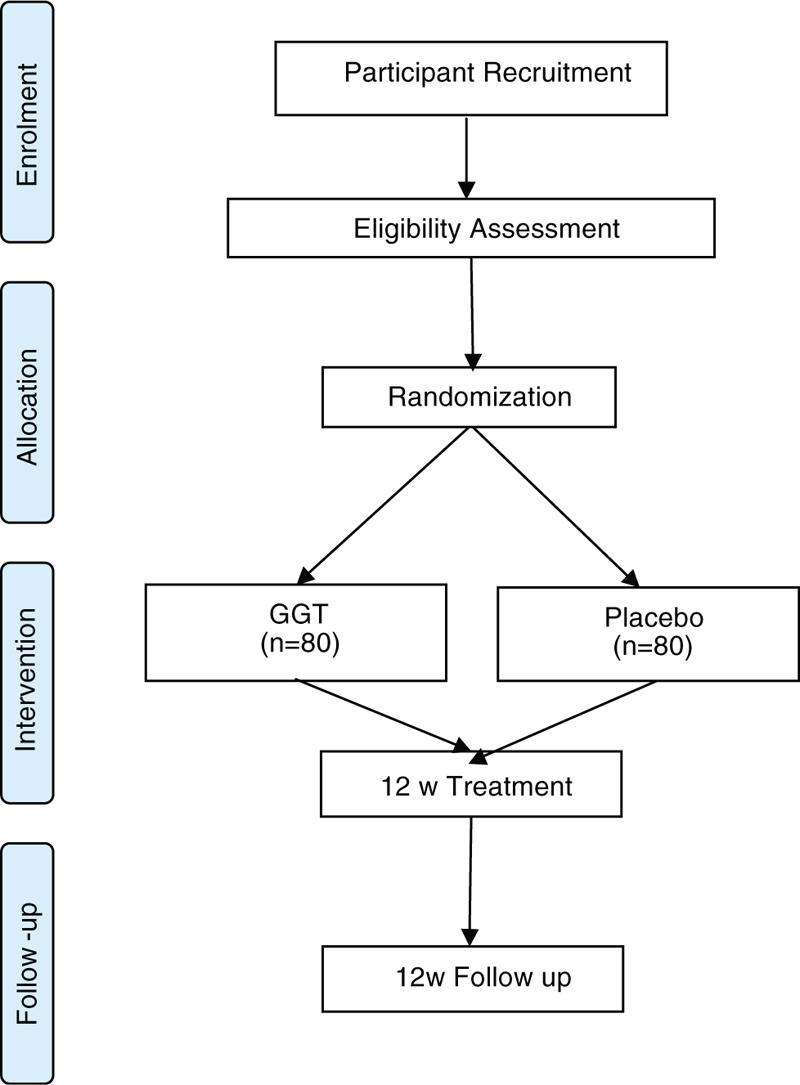
Study flow Diagram. From: Moher D, Liberati A, Tetzlaff J, Altman DG, The PRISMA Group (2009). Preferred Reporting Items for Systematic Reviews and Meta-Analyses: The PRISMA Statement. PLoS Med 6(7): e1000097. doi:10.1371/journal.pmed1000097. For more information, visit www.prisma-statement.org.

This will be a randomized, double-blinded, multi-center, placebo-controlled clinical trial that will be performed at 5 sites: Gachon University Gil Oriental Medical Hospital, Dongkook University Korean Medicine Hospital in Ilsan, Wonkwang University Guangju Medical Center, Dongsin University Oriental Hospital, and Kyunghee University Medical Center. The entire duration of trial will be approximately 24 weeks from the screening visit. The protocol design is based on the Consolidated Standards of Reporting Trials guidelines and Standard Protocol Items: Recommendations for Interventional of Trials Checklist.^[22,23]^ A schematic flow of the study is shown in Figure 1

### 3.1 Recruitment

The recruitment will be through a trial site bulletin board advertisement containing the following items: title, aim, duration, eligibility criteria, and intervention information of the trial. We will allow eligible participants to contact the trial staff for screening eligibility session voluntarily.

### 3.2 Participants

#### 3.2.1 Inclusion criteria

(1) Female 19 to 65 years’ old
(2) The subject must meet at least one of the following requirements;
  (2.1) Body mass index (BMI) of 30 kg/m^2^ or more;
  (2.2) BMI between 27 and 29.9 kg/m^2^ and either diagnosed by noninsulin-dependent diabetes mellitus (T2DM) and currently taking prescription medicine or with a fasting blood glucose level >126 mg/dL at the screening visit^[24]^
  (2.3) BMI between 27 and 29.9 kg/m^2^ and either diagnosed with hyperlipidemia and currently taking prescription medicine or has a total cholesterol level ≥200 mg/dL or TG ≥150 mg/dL at the screening visit^[25]^
(3) Consents to a calorie controlled diet during the trial
(4) Agrees to complete the written informed consent form

#### 3.2.2 Exclusion criteria

(1) Subjects who have a history of allergic reactions to the ingredients of the investigational drugs.
  (1.1) T2DM patients who have experienced an increase in blood glucose concentration by ephedrine
  (1.2) Have experienced rash, redness, or itching
  (1.3) Often experience fatigue due to hyperhidrosis
  (1.4) Have experienced other allergic reactions
(2) Subjects who lost 10% of body weight within 6 months.
(3) Subjects who have stopped smoking in the past 3 months or have irregular smoking habits
(4) Subjects with endocrine diseases associated with weight gain, such as hypothyroidism and Cushing syndrome
(5) Subjects with cardiovascular diseases (heart failure, ischemic chest pain, myocardial infarction, stroke or temporary ischemic cardioplegia)
(6) Patients with uncontrolled hypertension despite use of antihypertensive drugs (systolic blood pressure >145 mm Hg or diastolic blood pressure >95 mm Hg)
(7) Subjects with uncontrolled diabetes despite of drug treatment (fasting blood sugar test >7.8 mmol/L [140 mg/dL])
(8) Subjects with severe renal impairment (serum creatinine >2.0 mg/dL) or diagnosed with edema or dysuria
(9) Patients with severe liver failure (2.5 fold of upper limit of the normal range in alanine aminotransferase [ALT], aspartate aminotransferase [AST], alkaline phosphatase [ALP])
(10) Subjects with frequent gastrointestinal discomfort (loss of appetite, stomach pain, nausea, vomiting, etc) or who have a history of eating disorders such as anorexia (KEAT-26 <20)^[26]^
(11) Use of drugs that may have an effect on weight within last 3 months (appetite suppressant, laxative, oral steroid, thyroid hormone, amphetamine, cyproheptadine, phenothiazine or any medications can affect absorption, metabolism, excretion)
(12) Use of central nervous system stimulants for weight loss
(13) The use of medications that can increase blood pressure or heart rate, such as, decongestants, cough, cold, allergy treatments that include the ingredients of phenylpropanolamine, ephedrine, pseudoephedrine within the past week
(14) Subjects with diseases that can cause hypokalemia (hypomagnesemia, Bartter syndrome, Gitelman syndrome, diseases that can cause high aldosteronism, etc) or have cardiac dysrhythmia
(15) Subjects for whom the measures of anthropometric dimensions are difficult to obtain due to anatomical change such as resection
(16) History of weight loss surgery, cholelithiasis, narrow-angle glaucoma, malignant tumor, or lung disease
(17) Patients with a psychological history or who are currently suffering from and/or taking medications for the following diseases: severe depression, mania, bipolar disorder, schizophrenia, epilepsy, alcoholism, anorexia, and bulimia
(18) Subjects deemed unsuitable for the researcher's clinical study
(19) Any women who may be pregnant do not agree to appropriate contraception (birth-control pill, hormone implant, Intrauterine Device [IUD], etc) during study period
(20) Use of other investigational product (IP) or procedure within the last 1 month

### 3.3 Subject withdrawal or termination criteria

The criteria for subject withdrawal or termination are as follows: occurrence of a serious adverse event (SAE) that subjects are unable to tolerate; less than 70% drug compliance rate; use of any forbidden treatment during the trial that could impact on the study result; uncooperative subject; subjects who cannot follow up (FU); withdrawal of consent; site investigator's decision to terminate the process for the sake of the participant's health. Also, participants who are withdrawn after randomization will be followed up to examine outcomes.

### 3.4 Sample size

Given that there are no previous studies examining GGT for the treatment of weight loss, we referred to other weight loss research to estimate the pooled standard deviation of both groups^[3–9]^ for the sample size calculation.^[27]^ According to the ratio of test group: control group = 1:1, class I error α was 0.025 (1 side), class II error β was 0.2 (80%), and effect size set as a moderate effect (0.5) to estimate the sample size, 80 participants are required for each group. Considering the possibility of missing data and attrition (20% in total), we will recruit a total of 160 participants: 80 in the experimental group and 80 in the placebo group.

### 3.5 Randomization and blinding

In this study, the web-based randomization system (Institute of Safety and Effectiveness Evaluation for Korean medicine [ISEE] version) will be used to assign the randomization number at visit 2. Once a subject has passed the screening assessment, site clinical research coordinator or research assistants will contact the subject to arrange the next visit. At the enrollment visit, the subjects will be reassessed based on the eligibility criteria and distributed to 1 of 2 groups with a 1:1 allocation ratio web system. Then the site pharmacist will provide an IP with the same random number of assigned numbers to the subjects. All researchers, enrolled participants, and statisticians will be blinded throughout the study period unless a subject experiences SAEs warranting a code-break procedure in the case of emergency medical treatment.

### 3.6 Study procedures

After completing the informed consent form, subjects will be screened to confirm their criteria for eligibility. If subjects are identified as a candidate for this trial by site investigator, they will be required to register with the trial within 7 days after screening day. Once they register and are assigned by the randomization number, they will receive the investigational drugs based on their own randomization number and be scheduled for visits at the site every 4 weeks for efficacy and safety assessment for 12 weeks. At week 0 and 12, participants will receive the full battery of safety assessment via laboratory tests, and restricted safety assessment on liver and kidney function tests (ie, AST, ALT, γ-glutamyl transpeptidase, blood urea nitrogen, Cr, Na +, K +, CI-, lactic acid dehydrogenase, creatine kinase) at dosing period (week 4 and 8) for routine check-ups. After treatment ends, there will be 3 follow up (FU) visits every 4 weeks. At week 16 (FU1), participants will receive the full set of check-ups at screening visit for evaluation of the overall efficacy and safety of IP. The last 2 FUs (week 16, 20) participants will be assessed for any discomfort after cessation of IP. We will also collect data regarding individuals’ disease-specific cost and quality of life using a separately developed questionnaire and European Quality of life 5 Dimension (EQ-5D) for cost and effectiveness evaluation at each visit. During the study period, participants will be asked to adhere to a calorie-restricted diet and maintain a daily food log for self-calorie monitoring and will be instructed to avoid medications that may affect weight loss or the study results. The detailed study flow shown in Table 1.

**Table 1:**
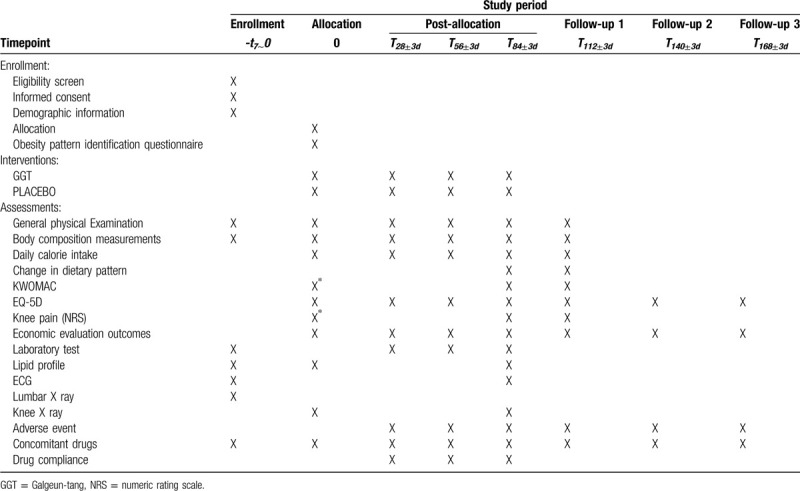
Study flow of the trial.

### 3.7 Interventions

In this trial, we plan to evaluate the efficacy and safety of GGT compared with placebo, given that there is currently no previous research on the use of GGT for antiobesity among patients with risk factors of metabolic syndrome. Both GGT and the placebo pills will be produced by the Hanpoong Pharm & Foods Co., Ltd. (Jeonju, Republic of Korea), and be ensured to be consistent in appearance, shape, and odor.

GGT is a traditional herbal formula which includes *Ephedrar Herbar*, *Paeoniae Radix*, *Zizyphi Fructus*, *Puerariae Radix*, *Cinnamomi Ramulus*, *Glycyrrhizae Radix et Rhizoma*, and *Zingiberis Rhizoma*.^[28]^ In this trial, we employed tablet materials, Beatcol Tab. which is an over the counter drug, based on the traditional Korean medicine prescription of GGT for improving drug compliance and convenience of portability.^[29]^

The placebo will consist of corn starch, microcrystalline cellulose, croscarmellose sodium, and coloring agents. Both groups will be administered the tablets orally with water between meals, 3 times a day for 12 weeks. When IP issues arise the site pharmacist will educate regarding the IP dosing method, possible side effects, next visit for IP compliance assessment to prevent dropouts, and protocol violations.

### 3.8 Outcomes

#### 3.8.1 Primary outcome measurement

The primary outcome is to assess the mean changes in body weight between the GGT and placebo groups from baseline to after 12 weeks of treatment.

#### 3.8.2 Secondary outcome measurements

##### 3.8.2.1 Body composition measurements

All patients will be measured for body fat percentage, fat mass, and BMI by using 8 tactile electrodes according to the manufacturer's instructions (InBody770 . Biospace Co. Ltd, Seoul, Korea) at each visit until FU1.^[30]^ The average changes in body fat percentage and fat mass from baseline to after treatment will be calculated.

##### 3.8.2.2 Anthropomorphic measurements

Waist circumference (WC), hip circumference (HC), and waist-hip ratio (WHR) will be evaluated at V2 to FU1. The circumferences of certain body regions will be measured by using a flexible nonstretchable tape measure while avoiding compressing. The specific measurement points include: WC will be measured at the smallest circumference between the ribs and the iliac crest and HC will be measured at the most protuberant area of hip, WHR will be estimated by dividing WC by HC.^[31]^ The average changes in both circumferences and WHR from baseline to after treatment will be calculated.

##### 3.8.2.3 Obesity screening laboratory tests

Serum glucose and lipid profile (ie, TG, total cholesterol, HDL cholesterol [HDL]) will be measured at screening and completion of treatment visits. Also, leptin and serum C-reactive protein will be evaluated at visit 2 and the drug discontinuation visit.

##### 3.8.2.4 Patient self-reported questionnaires

EQ-5D will be assessed at every visit to assess participants’ quality of life. EQ-5D is a tool that was developed to evaluate health-related quality of life and is widely used in the health care sector. The scores generally range from less than 0 to 1 and higher score indicates perfect health status.^[32]^ The average mean changes in the total score of EQ5D index at baseline and after treatment will be collected

Korean version of Western Ontario and McMaster Universities Questionnaire (K-WOMAC) and numeric rating scale (NRS) will be employed to measure any disabilities regarding knee function and pain due to obesity at the enrolment visit, end of treatment visit and FU 1 visit. The K-WOMAC is a validated patient-reported outcome that examines 3 components: pain (5 items), stiffness (2 items), and function (17 items). Each item score is anchored at 0 to 4 and a higher score indicates the worst condition of the knee.^[33]^ The average mean changes in the total score of K-WOMAC and NRS at each visit will be collected.

#### 3.8.3 Economic evaluation outcomes

An economic evaluation will be performed to assess the cost-effectiveness of the treatment. The primary economic endpoint will be cost per quality-adjusted life year gained, and secondary economic endpoints will cover such effectiveness parameters as cost per NRS. The primary analysis period will be 24 weeks and, if further estimation is needed, secondary analysis using regression-model or decision-modeling analysis to extrapolate cost and effect beyond the follow-up period will be performed.

#### 3.8.4 Other outcome measures

Measurement of knee condition in patients will be assessed using X-ray images based on the Kellgren–Lawrence classification from baseline between the GGT and placebo groups 12 weeks after treatment.^[34]^

#### 3.8.5 Safety outcomes

Observation of safety outcomes in this trial, vital signs, electrocardiogram, laboratory examinations (blood, urine routine tests, hematology, and biochemical test) and physical examination will be conducted during screening visits and the post-treatment phase. In the peri-treatment (visits 3, 4) period we will also perform laboratory tests for specific items, that is, ALT, AST, ALP, γ-glutamyl transpeptidase, blood urea nitrogen, creatinine, sodium, potassium, chloride, lactic acid dehydrogenase, creatine kinase for monitoring liver and kidney function. At each visit, the incidence of adverse events will be collected to assess the overall drug safety by asking the following question, “Were there any abnormal reactions since your last visit?.” If an incidence of adverse events is confirmed, collected data will be recorded in the case report form (CRF) thoroughly. The investigator will withdraw any participant experiencing a SAE and such patients will receive appropriate treatment. This will be reported to the site institutional review board (IRB) within 7 working days of withdrawal, according to the Korea Good Clinical Practice guidelines.

### 3.9 Statistical analysis

#### 3.9.1 Efficacy assessment

Baseline demographic data will be analyzed using a t-test for continuous data and a Chi-square test for categorical data. All outcome measures will be calculated as the mean changes between before and after treatments which will be compared using a paired *t*-test for intragroup analysis and a Student *t*-test for intergroup analysis. Any variables that are to be assessed more than twice during the trial will be analyzed by comparing the mean changes over time using a repeated-measures ANOVA test. Missing data will be distributed using the last-observation-carried-forward analysis method. There will be no interim analysis for any reason.

#### 3.9.2 Safety assessment

The safety datasets will include the participants who are to be administered GGT, or placebo at least once. The incidences of abnormal physical examination findings and self-reported adverse events will be compared using a Chi-square test. Liver function test, blood, and urine routine analysis data will be compared between the groups with a Student *t* test. Further, the data will be categorized into either normal or abnormal groups based on their respective laboratory reference ranges. Differences between the groups will be assessed with a Chi-square test.

#### 3.9.3 Economic evaluation

Effectiveness and utility measurements will be collected from the main comparative effectiveness randomized controlled trial. Quality of life will be measured by the EQ-5D instrument; quality-adjusted life years gained in both groups will be calculated using the area under the curve method.^[35]^ Treatment costs related to the clinical trial will be estimated by multiplying the number of treatment sessions by unit cost, and unit costs will be calculated using the data from the National Health Insurance and Medical Institution. If the total length of time is at least 12 months, the cost of the units will be standardized using the Korean monetary unit (won) value as of 2017 and applying a discount rate of 5% in accordance with Korean Health Insurance Review and Assessment Service economic evaluation guidelines. The analysis perspective of this study will be social and, in baseline analysis, representative values (eg, average) of study parameters will be used. All available distribution and representative values of parameter estimates will be applied in probabilistic sensitivity analysis. Cost and effectiveness (utility) will be analyzed as intention to treat and missing data will be imputed using multiple imputations.

### 3.10 Safety and data monitoring

This trial will be managed by Contract Research Organization, ISEE, Kyung Hee University. An assigned clinical research associate from ISEE will monitor this trial routinely in accordance with standard operating procedures. The brief monitoring plan is scheduled as follows: once the first participants complete the registration, completion of half of the planned enrolment of total, full enrolment for expected number of patients, case completion of last participant. To ensure the integrity of the quality of collected data, double data entry and data value range checks will be conducted. Auditing is not scheduled for the present study. Any other committee such as a coordinating center, steering committee, or endpoint adjudication committee will not be applicable to the present study.

### 3.11 Ethics and dissemination

This trial complies with the Declaration of Helsinki 2008 and the regulations of the Korea Good Clinical Practice. All items have been drawn from the World Health Organization Trial Registration Data Set (Version 1.3) (36). All of the IRBs at each study sites have reviewed and approved this clinical trial: Gil Oriental Medical Hospital, Gachon University (17-104), Dongguk University Oriental Medical Hospital (DUIOH 2017-10-004-006), Gwangju Oriental Hospital of Wonkwang University (2017/15-6), and Kyung Hee University Oriental Medical Hospital (KOMCIRB2017-10-044). This version of the current protocol is v1.7, and registered at clinicaltrials.gov on April 28, in accordance with the Standard Protocol Items: Recommendations for Interventional of Trials checklist (see Additional file 1). Once the protocol changes during the trial period, the modified version of the protocol must be approved by all IRBs of study sites before implementation. Written informed consent will be obtained before participation by the investigator (see Additional file 2). Moreover, we have contracted medical liability insurance for patient safety. The confidentiality of personal information will be ensured. Each participant will be assigned a trial registration number at enrolment. For the duration of the entire trial, data will be handled based on the subject's trial registration number. All records will remain secure in a locking storage cabinet or password-protected document files in a computer, both for the duration of the trial and after the trial has concluded. Only the investigators will retain the right to access the study data. The results of the study will be disseminated through scientific journals or scientific conference presentations. So far, no public access to the full protocol, participant-level datasets, or statistical code is planned.

## 4 Discussion

This will be the first multi-center, randomized, placebo-controlled trial, to the best of our knowledge, exploring the efficacy and safety of GGT among obese female patients with or without various risk factors of metabolic syndrome. Although findings from our preclinical experiments have indicated that GGT has significant antiobesity effects on a high-fat diet-induced obese mouse model,^[20]^ no previous studies have examined GGT for the treatment of overweight humans with or without metabolic risk factors. Thus, this study is necessary to investigate the efficacy and safety of GGT to establish well designed methodological evidence and promote the strength of Korean herbal medicine.

There are several limitations to the proposed study. First, although we will obtain the enrolled participants’ agreements on restricted low-calorie diet and request that they keep a daily calorie diary for self-evaluation of their daily calorie intake, their daily food intake will not be externally restricted. Thus, we will perform frequent researcher phone monitoring of participants to additionally assess their diet control success. Second, due to limited funding resources, a short-term drug administration period has been employed, with a small sample size; thus, a larger, long-term trial is required to confirm the impact of GGT on long-term weight and management of metabolic risk factors.

In conclusion, this pilot study will provide valuable evidence for the methodology and determination of the larger trial feasibility of GGT as a treatment for obesity among female patients with or without metabolic risk factors in Korea. Moreover, the results of this trial may help to promote the strengths of Korean medicine in the future. Apendix_ICF: Written informed consent will be obtained before participation by the investigator.

## Author contributions

**Conceptualization:** Hojun Kim, Jin-Bong Choi, Young-Dal Kwon, Won-Seok Jung, Bo-Hyoung Jang.

**Data curation:** Bo-Hyoung Jang.

**Funding acquisition:** Yun-Kyung Song, Seong-Gyu Ko.

**Investigation:** Hojun Kim, Jin-Bong Choi, Young-Dal Kwon, Won-Seok Jung, Yun-Kyung Song.

**Methodology:** Youme Ko.

**Project administration:** Yun-Kyung Song.

**Resources:** Seong-Gyu Ko.

**Supervision:** Seong-Gyu Ko.

**Validation:** Bo-Hyoung Jang.

**Writing – original draft:** Youme Ko, Hyun-Ju Kim.

**Writing – review and editing:** Bo-Hyoung Jang, Yun-Kyung Song.

## Supplementary Material

Supplemental Digital Content
