# Supplementary material for: Exploring the efficacy and safety of herbal medicine on Korean obese women with or without metabolic syndrome risk factors: A study protocol for a double-blind, randomized, multi-center, placebo-controlled clinical trial
Source: Medicine (Baltimore). 2020 Jul 10;99(28):e21153. doi: 10.1097/MD.0000000000021153 (PMC7360330; doi:10.1097/MD.0000000000021153)
Supplement: Supplemental Digital Content [file medi-99-e21153-s001.pdf]

[Appendix] Informed Consent Form (ver 1.5)

Study Title: Exploring the Efficacy and Safety of Herbal Medicine on Korean Obese Women with or without Metabolic Syndrome Risk Factors – A Study protocol for a double-blind, randomised, multi-center, placebo-controlled clinical trial

□ I have read the participant information sheet and I understood the purpose, methods, expected effect, possible risk, and information management collected in the study with a full explanation.
□ I have had the opportunity to ask questions about it and any questions that I have asked have been answered to my satisfaction.
□ I was also informed that I can withdraw the agreement and receive appropriate treatment if any adverse event occurs.
□ I understood the explanation about collecting, using and providing personal information.
□ I have a copy of this consent form and information sheet.
□ I have been given sufficient time to consider and I consent voluntarily to participate as a participant in this research.

Participant

Print Name ________________ Signature _________________ Date __________________

Legal representative (if necessary) (Relationship:)

Print Name ________________ Signature _________________ Date __________________

Witness (if necessary)

Print Name ________________ Signature _________________ Date __________________

Researcher/person taking the consent

Print Name ________________ Signature _________________ Date __________________
